# The Philadelphia Society of Veterinary Medicine

**Published:** 1891-11

**Authors:** E. H. Landes

**Affiliations:** General Secretary


*The Philadelphia Society of Veterinary Medicine.*—The Philadelphia Society of Veterinary Medicine was organized at Philadelphia, on May 15th, 1891, by the adoption of constitution and by-laws, and the election of the following officers: President, Dr. Wm. H. Ridge; Vice-President, Wm. Birch, V. S.; General Secretary, E. H. Landes, V. M. D.; Recording Secretary, J. J. Maher, V. M. D.; Librarian, Howard Felton, V. M. D. Executive Committee: S. J. J. Hargar, V. M. D., G. R. Hartman, V. M. D., W. L. Zuill, M. D., V. S., Howard Felton, V. M. D. The object of the society is the reading of papers and the discussion of all subjects pertaining to veterinary and comparative medicine. The society starts with a strong and vigorous membership and has every promise of leading a useful and successful career.

The society held its first regular meeting on September 23, 1891, at the Veterinary Department of the University of Pennsylvania. Dr. W. H. Ridge, the president in the chair, and the following members and visitors were present: members—Drs. H. D. Entirkin, A. E. Conrow, M. E. Conrad, Leonard Pearson, C. H. Magili, Robert Formad, E. Bunting, Wm. L. Zuill; E. Muir, S. J. J. Hargar, E. H. Landes; visitors—John Marshall, M. D., and Dr. Kramer.

The subject that held the attention of the meeting was: "Tuberculosis and Koch Lymph as a Diagnostic Agent in Cattle," the report of the Tuberculosis Commission of the Veterinary Department of the University of Pennsylvania, was read by Dr. Wm. L. Zuill. Dr. Leonard Pearson gave a synopsis of the experiments that were conducted in Europe for the purpose of determining the diagnostic value of the lymph, and it was found that the results obtained were the same as those of the University Commission. The subject was thoroughly discussed by Drs. Zuill, Ridge, Formad, Hargar, and others, and it was agreed that the lymph did possess considerable diagnostic value.

The secretary was instructed to provide a permanent place of meeting, at the College of Physicians, if possible.

E. H. Landes, *General Secretary.*

